# Aprosopia/holoprosencephaly in a stillborn puppy: when the face predicts the brain

**DOI:** 10.1080/23144599.2021.1897740

**Published:** 2021-03-26

**Authors:** Clairton Marcolongo Pereira, Tayná B. Silva, Laiz Zaché Roque, Bárbara Barros, Luiz Alexandre Moscon, Ana Lucia Schild, Claudio S. L. Barros, Leonardo Schüler-Faccini, Lavinia Schuler-Faccini

**Affiliations:** a Faculdade de Medicina Veterinária, Centro Universitário do Espírito Santo (UNESC), Colatina, ES, Brazi; b Laboratório Regional de Diagnóstico, Faculdade de Veterinária, Universidade Federal de Pelotas (UFPel), Capão do Leão, Brazil; c Laboratório de Anatomia Patológica, Universidade Federal de Mato Grosso do Sul (UFMS), Campo Grande, MS, Brazil; d Departamento de Genética, Serviço deGenética Médica Populacional (INAGEMP), Universidade Federal do Rio Grande do Sul (UFRGS), Porto Alegre, RS, Brazil

**Keywords:** Dog, central nervous system, congenital defects, aprosopia, holoprosencephaly

## Abstract

In a litter of three puppies, one was stillborn and had facial and brain defects. Fusion of the maxilla and mandible and absence of the face were observed. The forebrain (telencephalon and the diencephalon) was reduced in size and fused, and the telencephalic longitudinal fissure, olfactory bulbs, and optic nerves were absent (Figures 6 and 7). Lissencephaly was observed in the telencephalon and cerebellum. A diagnosis of aprosopia/holoprosencephaly was made.

## 1. Introduction

The central nervous system is a tubular structure that originates from the neural plate, a proliferation of ectodermal epithelial cells, referred to as the neurectoderm, located dorsal to the notochord along the axis of the embryo. The neural plate invaginates along this axis, forming a groove until the lateral extremities of the original plate, the neural folds, meet centrally and fuse over the neural groove to form a neural tube and canal. The rostral end of the neural tube develops and produces three vesicles, from rostral to caudal: the prosencephalon, mesencephalon, and rhombencephalon. The prosencephalon (forebrain) becomes subdivided into the diencephalon ventro-medially and telencephalon laterally and dorsally, forming the cerebral hemispheres. The rhomoboencephalon forms the cerebellum, pons and medulla oblongata [1].

Holoprosencephaly (HPE) is a malformation characterized by failure of the forebrain to separate into two cerebral hemispheres and incomplete cleavage or non-separation of midline structures. The degree of this lack of separation varies depending on the severity of the malformation and is classified as alobar, semi-lobar, and lobar [2].

Alobar HPE is the most severe form of the malformation, characterized by brain hypoplasia with complete lack of separation of the cerebral hemispheres, a single lateral ventricle, fusion of the midline structures and aplasia of the olfactory bulbs, corpus callosum, septum pellucidum and septal nuclei. Semi-lobar HPE is an intermediate form of this malformation with less severe cerebral hypoplasia than the alobar form. The rostral cerebral hemispheres fail to separate but the occipital lobes are distinct. The olfactory bulbs, septal nuclei, rostral corpus callosum and septum pellucidum are absent. In lobar HPE, the less severe form of HPE, there were an almost normal brain size, and a separation of the cerebral hemispheres except at the most rostral areas. The olfactory bulbs may be present but hypoplastic, the cranial portion of the corpus callosum absent or hypoplastic, and there is often heterotopic gray matter in the roof of the ventricle [2,3].

A newer subtype of HPE, termed aventriculi, is characterized by failed ventricular system development and has been described in one dog [4].

HPE, in its most severe form, is associated with skull and facial defects, such as aprosopia, microcephaly, cyclopia, proboscides, and medial cleft lip and palate. Aprosopia, characterized by congenital absence of most or all of the face, is a rare malformation in any animal species. Sheep seem to be the most commonly affected by this developmental defect [5–8].

HPE occurs in humans with a frequency of approximately 1/8,000 births, including relatively mild forms compatible with postnatal life [9]. In dogs, holoprosencepalhy is a rare malformation with few reports in literature. Semilobar [10] and lobar [4,11,12] forms have been reported.

Other defects of the face and brain of dogs, such as cranioschisis, palatoschisis, anophthalmia, corpus callosum malformations, and otocephaly, have also already been described [10,11,13–15].

Environmental toxicity, teratogenic agents, and genetic mutations can compromise fetal development and result in congenital defects that can range from small or moderate abnormalities to severe anomalies incompatible with postnatal life [16]. The artificial selection of dogs through inbreeding to establish new breeds has also been associated with the appearance of some genetically determined congenital defects, such as brachycephaly [17] or Arnold-Chiari malformation [18].

In sheep, HPE can occur secondary to the ingestion of the alkaloid cyclopamine, which interferes with the signaling of the Sonic hedgehog (SHH) molecule [19]. Mutations in the SHH gene are associated with HPE in humans [6].

Craniofacial changes in humans and animals are also often associated with maternal infections during pregnancy. An example is congenital microcephaly caused by the Zika virus in children. In cattle, maternal infections with other flaviviruses have been described to result in a malformed phenotype similar to that of humans infected with the Zika virus [20]. This report aimed to describe a case of alobar HPE in a mixed-breed dog with lissencephaly and aprosopia.

## 2. Case description

A stillborn mixed-breed puppy was referred to the Veterinary Teaching Hospital of our university. It was part of a three-puppy litter from a three-year-old bitch, which was up to date on its vaccinations and had no history of receiving any medication. The bitch was vaccinated against rabies, distemper, parvovirus, canine infectious hepatitis, parainfluenza, coronavirus, *Leptospira* serovars Canicola and Icterohaemorrhagiae. No vaccines were administered during pregnancy. The other puppies in the litter were healthy and showed no lesions or clinical changes.

**Figures 1-2.**
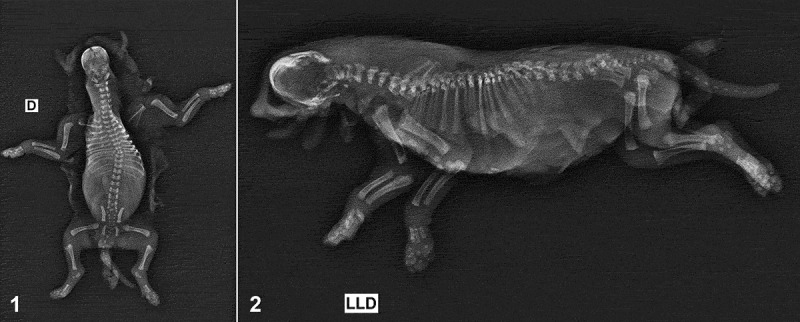
Dorsoventral and right lateral radiograph. The fusion of the maxilla and mandible can be observed

Radiographic examination revealed fusion of the maxilla and mandible (Figures 1 and 2).

**Figures 3-4.**
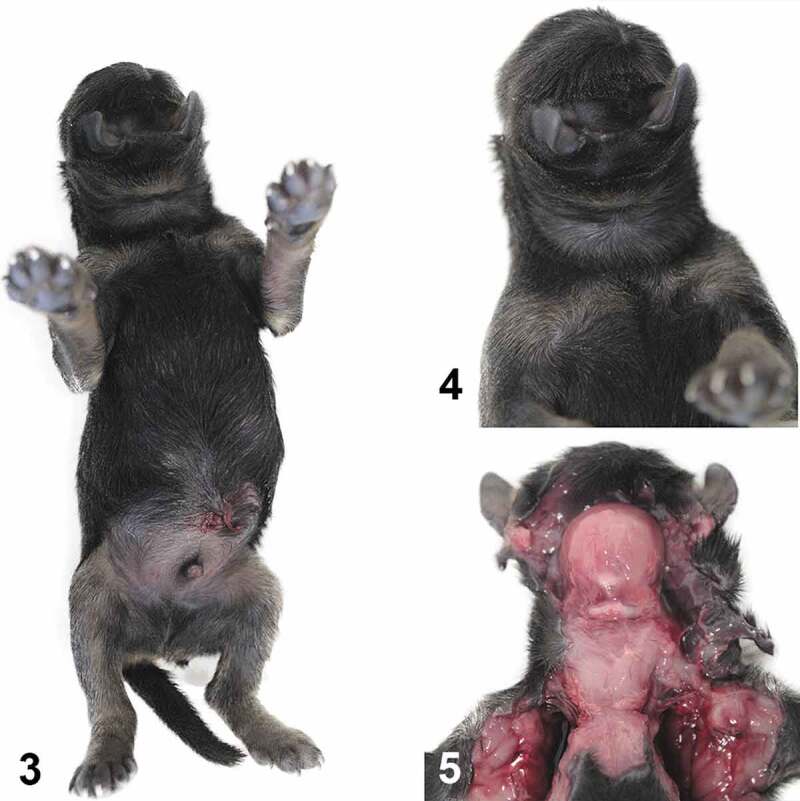
Unformed facial structure. The eyes, mouth, and nose are absent, and the ears are located ventrolaterally to the face. **Figure 5.**The muscles of the face, jaw, and maxilla are fused into a single muscle structure

On necropsy, the ocular globes, nose and mouth were absent and the external ears were located ventrolaterally to the face (Figure 3 and 4). The face, mandible and maxilla muscles were fused in a single structure (Figure 5). There was a bone callus substituting for the mandible and maxilla, the glottis was rudimentary, and the cranial portion of the trachea was incomplete.

**Figures 6-7.**
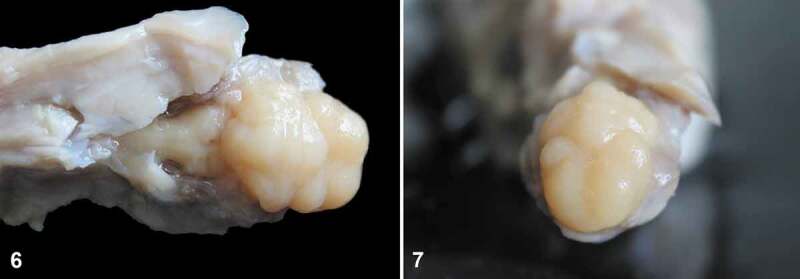
Ventral and rostral view of the brain. The forebrain is hypoplastic, the longitudinal telencephalic fissure is absent and there is no evidence of convolutions of the cerebral cortex

The forebrain (telencephalon and the diencephalon) was hypoplastic and fused into a single structure, and the telencephalic longitudinal fissure, olfactory bulbs, and optic nerves were absent (Figures 6 and 7). A single ventricle was observed. There were no cerebral or cerebellar cortex circumvolutions (lissencephaly). No further changes were observed. A diagnosis of alobar holoprosencephaly, lissencephaly, and aprosopia was made.

## 3. Discussion

This is the first report of alobar holoprosencephaly in dog, and similar facial malformations were found only in a dog with semilobar holoprosencephaly [10]. In both cases the animals presented lissencephaly. In a study in Shih Tzu dogs, the authors observed that lissencephaly occurred in three animals with also corpus callosum abnormalities but was not HPE [21]. Corpus callosum abnormalities have been sporadically reported in dogs, typically being an isolated abnormality or associated with holoprosencephaly [11].

In this report, the dam and other puppies were clinically healthy. However, it cannot be said with certainty that the dam was not exposed to any infectious or teratogenic agent that may have caused the malformation. In another study on HPE in a dog, the authors observed that only one of the puppies in a litter of five was affected, and they suggested that the malformation was inherited in an autosomal recessive manner [10].

## 4. Conclusion

In conclusion, this case is an example of the critical relationship between craniofacial and central nervous system development. HPE should be included in the differential diagnosis when facial and neurological abnormalities are observed in dogs.
